# Mutant p53: it’s not all one and the same

**DOI:** 10.1038/s41418-022-00989-y

**Published:** 2022-03-31

**Authors:** Margaret C. Kennedy, Scott W. Lowe

**Affiliations:** 1 Cancer Biology and Genetics Program, Sloan Kettering Institute, Memorial Sloan Kettering Cancer Center, New York, NY 10065 USA; 2 Louis V. Gerstner, Jr. Graduate School of Biomedical Sciences, Memorial Sloan Kettering Cancer Center, New York, NY 10065 USA; 3 Howard Hughes Medical Institute, New York, NY 10065 USA

**Keywords:** Cancer genetics, Tumour-suppressor proteins

## Abstract

Mutation of the *TP53* tumor suppressor gene is the most common genetic alteration in cancer, and almost 1000 alleles have been identified in human tumors. While virtually all *TP53* mutations are thought to compromise wild type p53 activity, the prevalence and recurrence of missense *TP53* alleles has motivated countless research studies aimed at understanding the function of the resulting mutant p53 protein. The data from these studies support three distinct, but perhaps not necessarily mutually exclusive, mechanisms for how different p53 mutants impact cancer: first, they lose the ability to execute wild type p53 functions to varying degrees; second, they act as a dominant negative (DN) inhibitor of wild type p53 tumor-suppressive programs; and third, they may gain oncogenic functions that go beyond mere p53 inactivation. Of these possibilities, the gain of function (GOF) hypothesis is the most controversial, in part due to the dizzying array of biological functions that have been attributed to different mutant p53 proteins. Herein we discuss the current state of understanding of *TP53* allele variation in cancer and recent reports that both support and challenge the p53 GOF model. In these studies and others, researchers are turning to more systematic approaches to profile *TP53* mutations, which may ultimately determine once and for all how different *TP53* mutations act as cancer drivers and whether tumors harboring distinct mutations are phenotypically unique. From a clinical perspective, such information could lead to new therapeutic approaches targeting the effects of different *TP53* alleles and/or better sub-stratification of patients harboring *TP53* mutant cancers.

## A long history

p53 was first discovered over 40 years ago in complex with the SV40 large T antigen in virally transformed cells [1, 2]. Although it was first classified as an oncogene, possibly because the initial studies inadvertently utilized a mutant p53 cDNA, it was later reclassified as a tumor suppressor gene after additional studies demonstrated that it could suppress growth and oncogenic transformation of cultured cells [3, 4]. In vivo studies of p53 null mice corroborated the in vitro data: while p53 null mice are developmentally normal, they ultimately develop tumors with nearly 100% penetrance [5].

In its essence, p53 is a stress-responsive transcription factor. Upon activation in response to a diverse array of stressors, the tetrameric form of the protein binds to DNA in a sequence-specific manner [6]. Once bound to DNA, p53 activates a range of antiproliferative programs, as well as the E3 ligase MDM2 to create a negative feedback loop that ultimately leads to degradation of p53 (refs. [7–13]). Virtually all p53 mutants studied to date have lost the ability to bind to DNA, thereby impairing its function as a transcription factor, and it seems likely that loss of this molecular function largely explains its role in tumor formation [14]. Owing to disruption of the p53-MDM2 negative feedback loop, many p53 mutant proteins are stabilized, allowing them to engage in aberrant interactions with other cellular factors, potentially altering their function and leading to GOF phenotypes [15–17].

### The spectrum of *TP53* mutations is unique among cancer genes

The spectrum of *TP53* mutations in human tumors is remarkable for its diversity and tissue specificity. There is strong enrichment for mutations in the DNA binding domain (DBD). DBD mutations are predominantly missense (~80%), including six “hotspot” codons (R175, R213, G245, R248, R273, and R282), which account for ~25% of all *TP53* mutations. In contrast, mutations that occur outside of the DBD are more likely to be nonsense or truncating mutations (~67%) than missense mutations [18, 19]. In addition, beyond acquiring a *TP53* mutation in one allele, most tumors lose the second allele by deletion or copy neutral loss of heterozygosity [20, 21]. The extent to which this allelic variation is a consequence of the underlying mutational mechanisms or biological selection for functionally meaningful mutations—or both—remains incompletely understood. Most of the hotspot residues contain methylated CpGs, which are five times more likely than unmethylated cytosines to undergo spontaneous deamination producing the observed C > T mutation [19, 22]. Since the majority of *TP53* mutations are not encoded in the germline, the observed frequency of *TP53* mutations may also be influenced by the immunogenicity of the mutant protein, with those least likely to be surveilled producing a greater advantage [23, 24]. Finally, different mutants may have different biological potencies, with more recurrent alleles having more potent oncogenic effects.

The frequency of *TP53* mutation varies greatly between different tumor types. *TP53* is mutated in more than 90% of ovarian cancers whereas less than 15% of acute myeloid leukemias (AML) have *TP53* mutations, suggesting that there may be some tissue-specific requirements for loss of wild type or gain of mutant p53 functions [25]. Further support for the tissue specificity of mutant p53 function comes from studies of Li Fraumeni Syndrome patients. Li Fraumeni patients have inherited a germline *TP53* mutation and therefore have a high risk of developing cancer [26–28]. Intriguingly, a Li Fraumeni cohort from southern Brazil harbors a founder mutation, R337H, that gives rise to pediatric adrenal cortical carcinoma at a much higher frequency than other mutations. These observations hint that the mutant R337H protein may have gained a function that specifically promotes tumor formation in the kidney [29]. That said, some aspects of genetic background may also contribute to this specificity, since an analysis of the MSK-IMPACT dataset indicates that the one patient with a germline *TP53*^*R337H*^ allele presented with prostate and stomach cancer, and none of the 15 patients with acquired *TP53*^*R337H*^ mutations develop adrenal carcinoma [30, 31].

If *TP53* mutations across tumor types produce a heterogeneous functional output, it is possible that mutants differ in the extent to which they inactivate wild type p53, serve as a DN, and/or produce oncogenic GOF activities that promote cancer beyond inactivating p53. An overhwhelming body of evidence suggests that p53 must be inactivated to promote tumorigenesis [32]. There is also no question that certain missense mutant proteins can have dominant negative activities [33]. Still, the fact that the vast majority of tumors ultimately inactivate the remaining wild type p53 allele implies that the DN effect might not be able to completely inactivate wild type p53 tumor suppressive function. Indeed, the composition of tetramers in a heterozygous cell range from fully wild type to fully mutant, and the strength of the DN correlates with increasing numbers of mutant subunits in the tetramer [34]. Alternatively, there may be selection for additional pro-oncogenic effects. Whether any or all *TP53* mutations have GOF activities, and whether these functions are biologically relevant, remains the subject of intense research. Below we discuss recent studies refuting or supporting the p53 mutant GOF hypothesis and refer the reader to other excellent reviews for a comprehensive summary of the data [7, 11, 35–37].

### Evidence for gain of function

The potential GOF effects of p53 mutant proteins have been the topic of debate for nearly 30 years. The first hint that p53 mutant proteins could have GOF activity came from Levine and colleagues, who showed that ectopic expression of certain *TP53* mutant alleles could activate the expression of a multi-drug resistance gene reporter while the wild type p53 could not. Different *TP53* mutants showed a range of activities, providing an early hint that not all *TP53* mutations function equivalently [38]. Later, two other groups found that although mice engineered to have germline missense mutations (R175H and R273H) succumbed to cancer at a similar rate as p53 null, they displayed a broader tumor spectrum and a higher incidence of metastasis [39, 40]. These data were viewed as decisive evidence of a GOF effect.

Since these early studies, thousands of more recent papers provide additional evidence that mutant p53 can influence biological functions such as metastasis, stemness, epithelial to mesenchymal transition, and many others [35–37]. Despite this, the field has yet to converge on a cohesive model to explain these provocative results. Although many of the proposed GOF mechanisms involve mutant p53 interacting with other transcriptional regulators to induce changes in gene expression, a staggering number of effectors have been identified [41]. Not only do different *TP53* mutations appear to have different GOF capabilities, but even the same mutant appears to act through distinct mechanisms depending on the context (see for example [42–44]), suggesting that genetic background may be responsible for some of the inconsistencies. This complexity is even more remarkable given that most studies only characterize one or a few mutants. It is therefore not surprising that some investigators have begun to question whether p53 GOF plays a meaningful role in cancer biology, and they are beginning to take a comprehensive approach to address this question.

### A more systematic approach

Recently, two groups conducted saturation mutagenesis screens by exogenously expressing a library of mutant p53 cDNAs in cancer cell lines. Giacomelli and colleagues conducted their screen in isogenic *TP53*^*+/+*^ and *TP53*^*−/−*^ human A549 lung adenocarcinoma cells while Boettcher et al. did the same in a K562-*TP53*^wild type^ CML cell line which expressed a GFP reporter at the *CDKN1A* locus [33, 45]. Both groups found that DBD mutants showed a dampened senescence response following treatment with the MDM2 inhibitor Nutlin-3a. In contrast, cells harboring N- or C-terminal mutations in p53 maintained an intact senescence response. Together, these reports provide strong evidence that, at least in the in vitro assay used, p53 DBD mutants, but not other mutants, can act as DN proteins.

Boettcher et al. also conducted RNA sequencing of K562 cells that express wild type p53 or a hotspot mutation in the absence of the wild type [45]. They found that there was little variation between the genes that were downregulated by different mutants, leading the authors to conclude that the primary function of mutant p53 is to inactivate wild type transcriptional programs. However, these data were also consistent with a potential GOF role for the mutants in this system: there is a subset of genes that were activated by the mutants to a greater extent than the wild type, though the specific genes varied between mutants.

To assess the potential of mutant p53 to function as a DN in vivo, Aubrey et al. conducted competitive transplantation assays with hematopoietic stem and progenitor cells derived from three different mouse models (*Trp53*^*+/−*^, *Trp53*^*−/−*^, and *Eμ-Myc;Trp53*^*+/+*^) that express p53 cDNAs harboring select DBD mutations [32]. They found that mutant p53 cDNAs could dramatically accelerate tumorigenesis in the *Trp53*^*+/+*^ background but had no impact on the already rapid tumorigenesis that occurs in the *Trp53*^*−/−*^ background. In the *Trp53*^*+/−*^ background, the *Trp53* mutant cDNAs apparently confer a selective advantage not by accelerating tumorigenesis but rather by biasing the tumors to a myeloid differentiation state.

The above results reiterate the potential role for a DN effect at early stages of tumorigenesis prior to the ‘second hit’ but did not support a GOF activity. Still, the latter conclusion is based largely on the lack of tumor acceleration when p53 mutant cDNAs are introduced into the p53 null background, an observation that is potentially confounded by the potential presence of pre-existing tumor cells in the transduced *Trp53*^*−/−*^ population. Nonetheless, the same group has provided orthogonal support for this notion by showing that CRISPR-mediated disruption of mutant *TP53* in a range of human cancer cell lines has no effect on proliferation or survival in vitro or following transplantation into immunocompromised animals [46].

### Irreconcilable differences

Collectively, the above studies elegantly confirm what we have known for some time—that *TP53* mutations both impair wild-type p53 functions and can act, to varying degrees, as dominant negative proteins. However, their impact relates less to what was observed compared to what was not: any meaningful oncogenic GOF for mutant p53. How then, can we reconcile these systematic studies with the countless peer-reviewed studies showing p53 can have GOF oncogenic effects, often in well-controlled isogenic settings?

In principle, there could be some underlying feature about mutant p53 that produces real biology but may not be selected for during tumorigenesis. Another possibility is that the assays used do not fully capture the range of settings where p53 GOF plays a role. Most in vitro studies relied heavily on profiling the proliferation of cancer cells, which is a too limited readout to assess the function of a protein that is known to regulate many different cellular processes. In support of the need to systematically conduct assays that capture the breadth of p53 functions, one group found that hotspot mutations did not proliferate any better than other mutants in 2D or 3D cell culture, yet they were robustly selected for upon transplantation into nude mice, implying a GOF effect [47].

Although some of the studies described above did look at tumor initiation in vivo, many of the GOF activities affect aspects of cancer that may not be as relevant in the hematologic cancer models used. For example, the best characterized GOF activity of mutant p53 is its role in promoting metastasis [43, 48]. Furthermore, the tumor-promoting functions of mutant p53 may be influenced by the microenvironment. Indeed, a recent study showed that mutant p53 promotes tumor formation in the distal gut due to an interaction with gut microbiota, whereas the mutant was tumor suppressive in the proximal gut which has much lower levels of bacteria [49]. Moreover, recent reports have uncovered a role for mutant p53 in modulating immune surveillance. Both the R249S and the R175H mutants suppress the recruitment of T cells and NK cells while promoting the recruitment of pro-angiogenic M2 macrophages and neutrophils [23, 24].

It is hard to refute the plethora of technically sound studies that identify a GOF role for mutant p53. Because many of these studies have focused on only a few mutants, it has been extraordinarily difficult to sort out to what extent there is a shared GOF mechanism between different mutants in the same setting, and to what extent a mechanism is conserved between the same mutant in different settings. To that end, one solution to the DN vs GOF debate is to continue to systematically study a range of mutants, but expand the readout to a range of phenotypes, including ones in vivo, to capture both potential DN and GOF properties and any allelic variation. Thus, the saga continues.

### An alternative hypothesis

Whether one or more of the above hypotheses prove to be biologically relevant, it nonetheless remains difficult to conceptualize how a range of distinct mutant proteins could acquire such potent biological activities without some basis in normal physiology. As such, it is attractive to consider the possibility that some *TP53* mutants acquire pro-oncogenic activities through a ‘separation-of-function’ (SOF) rather than GOF mechanism. In principle, mutations that selectively retain the pro-proliferative or survival functions of wild type p53 (e.g. adaptation to metabolic stress) while disrupting the canonical tumor suppressive activities (e.g. apoptosis, senescence) might produce phenotypes that appear similar to a GOF (Table 1) (refs. [7, 37, 41]). As one example, cancer-associated recurrent exon 6 *TP53* truncating mutations mimic a naturally occurring, pro-proliferative splice variant, p53-psi, and can produce a metastatic phenotype that non-exon 6 truncations cannot [20]. Other reports hint at SOF in what might classically have been labelled GOF: for example, R248W, but not R175H, is able to promote cell survival in serine depleted conditions by activating the serine synthesis pathway and antioxidant response; other mutants retain wild type p53’s ability to suppress autophagy which promotes survival in hypoxic conditions [50, 51]. RNA sequencing data generated by Aubrey et al, though analyzed through the lens of the DN hypothesis, supports the existence of mutant p53 SOF at the transcriptional level: while many well-known p53 target genes were downregulated by expression of a mutant, about 40% of canonical targets were not. Furthermore, the hotspot mutations had higher expression of target genes that may be advantageous for tumor development than the other mutants [32]. While evidence for the SOF hypothesis remains sparse, if confirmed, could lead to a more predictable and unified model explaining *TP53* mutational variation and thus is worthy of further exploration.

**Table 1:** Select examples of mutant p53 separation-of-function.

Function	Pro- or anti-proliferative or survival	Loss or retention of WT function	Experimental evidence	Ref
DNA damage response	Pro	Loss	Mutants (V173M, I195S, R248Q, R273H, insG282) repress transcriptional program in *Eμ-Myc* lymphoma cell lines	[32]
Antioxidant response	Pro	Retain	R248W mutant in HCT116 cells retains MDM2-ATF4 antioxidant response to survive in serine and glycine depleted conditions	[50]
Autophagy	Pro	Retain	R175H, G245S, R248W, R249S, R273H retain the ability to suppress autophagy, which may promote genetic instability and pro-tumorigenic inflammation, among other oncogenic events	[51]
Chromatin remodeling	Pro	Retain	R273H mutant interacts with SWI/SNF in MDA-468 cells to increase pro-angiogenic *VEGFR2* expression	[52]
Metastasis	Pro	Retain	Exon 6 truncating mutations retain the pro-metastatic capabilities of p53-psi	[20]
Apoptosis	Anti	Retain	E180R mutant activates Puma to sensitize an *NRas*^*G12D*^*;AML1/ETO9a* AML mouse model to chemotherapy	[53]
Cell cycle arrest	Anti	Loss	Mutants (R175H, R273H, R273H/P309S) form a complex with NF-Y to upregulate pro-proliferative genes, resulting in aberrant cell cycle progression after DNA damage	[54]

### An important question to resolve

Achieving clarity in our understanding of how mutant p53 proteins influence cancer phenotypes would be of great benefit to the field and to the treatment of patients with tumors that harbor mutant p53. Currently, patients are stratified into wild type and mutant p53 during diagnosis; however, if there is substantial allelic variation, a better classification system would be to stratify patients by functional class of the mutant. One can imagine that this type of classification of mutant p53 may also reveal novel dependencies and therapeutic vulnerabilities such that in the future, patients may receive targeted therapy based on the functional class of the *TP*53 mutation.

## Supplementary information

Author contribution form
