# Supplementary material for: Mutant p53: it’s not all one and the same
Source: Cell Death Differ. 2022 Mar 31;29(5):983–7. doi: 10.1038/s41418-022-00989-y (PMC9090915; doi:10.1038/s41418-022-00989-y)
Supplement: Supplementary file 1 — Author contribution form [file 41418_2022_989_MOESM1_ESM.pdf]

DECLARATION OF CONTRIBUTIONS TO ARTICLE

ADMC

Manuscript Number:	Journal Name:	
CDD-22-0457	*Cell Death & Differentiation*	(the 'Journal')

Proposed Title of the Contribution:

Mutant p53: it's not all one and the same (the 'Contribution)

Author(s):

Margaret C. Kennedy and Scott W. Lowe (the 'Authors')

For all *CDD* articles, each person named as an author in the published version must be able to show he or she has contributed substantially to the article.

Authorship credit should be based on 1) substantial contributions to conception and design, acquisition of data, or analysis and interpretation of data; 2) drafting the article or revising it critically for important intellectual content; and 3) final approval of the version to be published. Authors should meet conditions 1, 2 and 3.

Any person who cannot be shown to have made a substantial contribution to the article cannot be listed as an author in the final version. The name of any person who is deemed to have made a minor contribution can, however, appear in the Acknowledgments section of the article.

Please complete the table below to indicate the contributions of all named authors to the manuscript.

Author Full Name:	Specification of Contribution to the Manuscript:
Margaret C. Kennedy	Conceived the idea and wrote the manuscript
Scott W. Lowe	Conceived the idea and wrote the manuscript

Please complete the table below to indicate the contributions of all named authors to the figures.

Figure 1:

Table 1. M.C.K. and S.W.L conceived of the idea. M.C.K. assembled the table.

Figure 2:

Figure 3:

Figure 4:

Figure 5:

Figure 6:

Signed for and on behalf of the Author(s):	Print Name:	Date:
	Scott W. Lowe	2/28/2022
